# Activation of the STAT3/microRNA‐21 pathway participates in angiotensin II‐induced angiogenesis

**DOI:** 10.1002/jcp.28564

**Published:** 2019-04-04

**Authors:** Li‐Yuan Chen, Xue Wang, Xiao‐Long Qu, Li‐Na Pan, Ze‐Yang Wang, Yong‐Hui Lu, Hou‐Yuan Hu

**Affiliations:** ^1^ Department of Cardiology Southwest Hospital, Third Military Medical University Chongqing China; ^2^ Department of Occupational Health Third Military Medical University Chongqing China

**Keywords:** angiogenesis, angiotensin II, atherosclerosis, miR‐21, STAT3

## Abstract

Angiotensin II (AngII) facilitates angiogenesis that is associated with the continuous progression of atherosclerotic plaques, but the underlying mechanisms are still not fully understood. Several microRNAs (miRNAs) have been shown to promote angiogenesis; however, whether miRNAs play a crucial role in AngII‐induced angiogenesis remains unclear. This study evaluated the functional involvement of miRNA‐21 (miR‐21) in the AngII‐mediated proangiogenic response in human microvascular endothelial cells (HMECs). We found that AngII exerted a proangiogenic role, indicated by the promotion of proliferation, migration, and tube formation in HMECs. Next, miR‐21 was found to be upregulated in AngII‐treated HMECs, and its specific inhibitor potently blocked the proangiogenic effects of AngII. Subsequently, we focused on the constitutive activation of STAT3 in the AngII‐mediated proangiogenic process. Bioinformatic analysis indicated that STAT3 acted as a transcription factor initiating miR‐21 expression, which was verified by ChIP‐PCR. A reporter assay further identified three functional binding sites of STAT3 in the miR‐21 promoter region. Moreover, phosphatase and tensin homolog (PTEN) was recognized as a target of miR‐21, and STAT3 inhibition restored AngII‐induced reduction in PTEN. Similarly, the STAT3/miR‐21 axis was shown to mediate AngII‐provoked angiogenesis in vivo, which was demonstrated by using the appropriate inhibitors. Our data suggest that AngII was involved in proangiogenic responses through miR‐21 upregulation and reduced PTEN expression, which was, at least in part, linked to STAT3 signaling. The present study provides novel insights into AngII‐induced angiogenesis and suggests potential treatment strategies for attenuating the progression of atherosclerotic lesions and preventing atherosclerosis complications.

## 1 INTRODUCTION

Atherosclerosis, cardiovascular disease with a high incidence, eventually results in acute cardiovascular events, such as stroke and myocardial infarction, which are caused by occlusive thrombosis due to rupture or erosion of atherosclerotic plaques (Hansson, Libby, & Tabas, 2015). Although numerous studies have focused on the pathophysiology of atherosclerosis, the exact underlying mechanism of plaque progression remains unclear, which has resulted in a lack of effective treatment. In addition to excessive cholesterol deposits, thin fibrous caps and aggravation of inflammation, intraplaque angiogenesis (IPA), and intraplaque hemorrhage (IPH) have been demonstrated to play critical roles in atherosclerotic plaque growth and destabilization (Michel, Virmani, Arbustini, & Pasterkamp, 2011). Upon stimulation, vascular endothelial cells (VECs) switch to a highly migratory and proliferative state that extends the pre‐existing vascular network. However, the mechanism by which these stimuli result in angiogenesis is not fully understood.

Angiotensin II (AngII), a potent vasoactive substance of the renin–angiotensin system, is believed to play a critical role in atherosclerotic plaque development. AngII has been reported to induce inflammatory cell accumulation and the proliferation of vascular smooth muscle cells by triggering the production of reactive oxygen species and upregulating the expression of adhesion molecules and chemokines (Daugherty & Cassis, 2004; Yamada et al., 2007). In addition, AngII also exerts growth factor‐like effects, which induces VECs proliferation, promotes IPA, accelerates atherosclerosis progression, and eventually leads to malignant cardiovascular events such as plaque hemorrhage and plaque rupture. A clinical trial also confirmed that AngII type 1 receptor (AT1R) antagonists reduced atheroma volumes in patients with coronary artery disease (Hirohata et al., 2010). Importantly, exposure to AngII has been shown to promote angiogenesis (Mai et al., 2014). Recently, AngII was found to induce human umbilical vein endothelial cell migration and proliferation (Si et al., 2018). Several other studies showed that low levels of AngII could induce angiogenesis, while high levels of AngII could induce apoptosis (Hoffmann et al., 2017; Lin et al., 2017). Further verifications are needed to clarify the angiogenic effect of AngII and understand the molecular mechanisms associated with such an effect.

MicroRNAs (miRNAs) are endogenous noncoding small RNA molecules (20–25 nucleotides) that regulate a wide range of physiological and pathological processes, including cell development, cell metabolism, cell aging, cell death, and other key biological processes. To date, multiple miRNAs, such as miRNA‐21 (miR‐21), miR‐155, miR‐210, and others, have been reported to regulate angiogenesis (Kir, Schnettler, Modi, & Ramakrishnan, 2018; Luo et al., 2017; L. L. Sun, Li, Lei, & Li, 2018). AngII has also been shown to modulate miRNA expression profiles in vitro and in vivo (Jin et al., 2012; Kim et al., 2014). Although the potential molecular mechanisms have not been fully elucidated, the proangiogenic miR‐21 has been proposed to be a potential target of AngII (Ning et al., 2017). Consequent activation of several growth‐promoting and antiapoptotic pathways, including PI3K, MAPK, and signal transducer and activator of transcription 3 (STAT3), are implicated in the stimulatory effects of AngII (P. K. Mehta & Griendling, 2007). Constitutive activation of STAT3 is strictly required for the maintenance of the AngII‐mediated phenotypic switch (Marrero, Fulton, Stepp, & Stern, 2004; Schmitz et al., 1998). The relationship between STAT3 signaling pathway and angiogenesis has been examined in the carcinogenic processes, but few studies have reported the role of this molecule in atherosclerotic plaque. Furthermore, the miR‐21 expression has been shown to be modulated by STAT3 signaling in certain other cell types and disease models, such as renal cell and chronic lymphocytic leukemia (Chen et al., 2018; Su et al., 2017). Nevertheless, whether the STAT3/miR‐21 pathway is involved in AngII‐induced angiogenesis requires confirmation. Thus, in the present study, we first explored the proangiogenic effect of AngII on endothelial cells (ECs) and then determined whether AngII treatment could induce STAT3 phosphorylation and miR‐21 expression. Moreover, we evaluated whether the modulation of the STAT3/miR‐21 pathway could affect AngII‐induced angiogenesis in vivo and in vitro, and the interaction of STAT3 and miR‐21 was analyzed by testing the binding of STAT3 to specific promoter sites of miR‐21. Furthermore, the downstream target of miR‐21 was also investigated in our study.

## 2 MATERIALS AND METHODS

### 2.1 Cell culture

Human microvascular endothelial cells (HMECs) were purchased from American Type Culture Collection (Manassas, VA). The cells were cultured in Dulbecco's modified Eagle's medium (DMEM; Gibco, Grand Island, NY) supplemented with 10% fetal bovine serum (Gibco) and 1% (vol/vol) penicillin/streptomycin (Beyotime Biotechnology, Beijing, China) and maintained in a humidified incubator at 37°C with 5% CO_2_. All experiments were performed using cells from passages 3–5.

### 2.2 HMEC proliferation assay

HMEC proliferation was measured using the Cell Counting Kit‐8 (CCK‐8; Dojindo, Kumamoto, Japan). HMECs were seeded in 96‐well plates (Costar, Corning, NY) at a density of 3 × 10^3^ cells/well and then treated with different concentrations (0, 10, 100, or 1,000 nM) of AngII (Millipore, Boston, MA) and Stattic (2 µM) in serum‐free DMEM for a period of 24 hr or transfected with miR‐21 mimic (30 nM), inhibitor (100 nM), and their respective NCs and allowed to grow for 24 hr at 37°C. After incubation for 2–4 hr, 10 μl of CCK‐8 solution was added to each well of the 96‐well plate, and the optical density value was measured by determining the absorbance at 450 nm with an Infinite M200 Microplate Reader (Tecan, Salzburg, Austria). For the EdU assay, the cells were treated with 20 μM EdU (RiboBio Co., Ltd., Guangzhou, China) and incubated for 2 hr at 37°C. The cells were fixed by incubation in 4% paraformaldehyde/phosphate‐buffered saline (PBS) solution for 30 min at room temperature followed by permeabilization with 0.5% Triton X‐100/PBS solution for 10 min. After three washes with PBS, the cells were stained with 100 μl of Apollo Dye Solution for 30 min. All cells were counterstained with DAPI staining solution to image the chromatin. Images were then captured using a fluorescence microscope. All experiments were performed in triplicate and repeated at least three times.

### 2.3 Wound‐healing migration assay

HMECs were seeded into 96‐well plates at 5 × 10^3^ cells/well and cultured to 80% confluence. A 600 µm‐wide wound scratch was generated in the center of all wells using a wound‐maker apparatus (Essen Bioscience, Ann Arbor, MI). The HMECs were washed twice with PBS and incubated in serum‐free DMEM containing different concentrations (0, 10, 100, and 1,000 nM) of AngII or Stattic (2 µM). For the transfection experiment, after 6 hr of transfection with hsa‐miR‐21 mimic (30 nM), hsa‐miR‐21 inhibitor (100 nM), and their respective NCs, a 24‐hr incubation with AngII was performed. Following wound scratching, images were obtained at 6 hr intervals from 0 to 24 hr using the IncuCyte ZOOM Live‐Cell Analysis System equipped with time‐lapse bright field microscopy (Essen BioScience). The data were also analyzed using IncuCyte ZOOM™ 2013A software (Essen BioScience) based on wound confluence, an integrated metric, which indicates the confluence of cells within the wound region.

### 2.4 In vitro Matrigel tube‐formation assay

An in vitro capillary‐like tube‐formation assay was performed as previously described (C. Sun et al., 2017). Briefly, a 96‐well plate was precoated with 60 μl of growth factor‐reduced Matrigel (BD Biocoat, Corning, NY) and incubated at 37°C for 1 hr to allow for polymerization. HMECs (1 × 10^4^) were seeded with different concentrations (0, 10, 100, and 1,000 nM) of AngII or Stattic (2 µM) in serum‐free DMEM. For the experiment with miRNAs, HMECs were seeded in 12‐well plates at 1.5 × 10^5^ cells/well and transfected with hsa‐miR‐21 mimic (30 nM), hsa‐miR‐21 inhibitor (100 nM), and their respective NCs followed by incubation with AngII (100 nM). HMECs were trypsinized 24 hr after the indicated transfections and then reseeded onto Matrigel‐coated 96‐well plates. After 12 hr of culture in serum‐free DMEM, the capillary formation was assessed microscopically at five random locations (×100 magnification). The number and length of capillaries were measured by Image‐Pro Plus 6.0 software.

### 2.5 Cell transfection

Before transfection, the indicated cells were cultured to 80% confluence. miR‐21‐5p mimic (30 nM), miR‐21‐5p inhibitor (100 nM), and their respective NCs were synthesized by RiboBio (RiboBio Co., Ltd.) and transfected into the HMECs using Lipofectamine 2000 (Invitrogen, Carlsbad, CA) according to the manufacturer's instructions.

### 2.6 Quantitative real‐time PCR (qRT‐PCR) analysis of miRNA

Small RNAs were extracted using the miRcute miRNA Isolation Kit (Tiangen Biotech Co., Ltd., Beijing, China) according to the manufacturer's instructions. The RNA concentration was determined using a NanoDrop spectrophotometer (Thermo Fisher Scientific, Waltham, MA). Small RNAs were used to synthesize complementary DNA (cDNA) by reverse transcription with a miRcute Plus miRNA First‐Strand cDNA Synthesis Kit (Tiangen Biotech Co., Ltd.) with a reaction system volume of 20 μl. The forward primer sequence was 5′‐TAGCTTATCAGACTGATGTTGA‐3′. The reaction conditions of reverse transcription were as follows: 42°C for 60 min and 95°C for 3 min. qRT‐PCR was performed using the SYBR^®^miRcute Plus miRNA qPCR Detection Kit (Tiangen Biotech Co., Ltd.). The reaction conditions of PCR were as follows: 95°C predenaturation for 15 min, followed by 40 cycles of 94°C denaturation for 20 s, 58°C annealing for 30 s, and a 72°C extension for 10 s. OpticonMonitor3 software (CFX96; Bio‐Rad Laboratories, Hercules, CA) was applied to analyze miR‐21 expression in HMECs. U6 small nuclear RNA was used as an internal control. The results of the qRT‐PCR analysis were determined based on the threshold cycle (*C*_t_), and the relative expression levels were calculated using the $2^{-\Delta \Delta C_{t}}$ method after normalization to the expression of the internal control gene.

### 2.7 Western blotting

After treatment, HMECs were harvested and homogenized with RIPA lysis buffer (Beyotime Biotechnology) containing protease inhibitor cocktail. Twenty‐five micrograms of isolated protein was subjected to 10% sodium dodecyl sulfate‐polyacrylamide gel electrophoresis and transferred onto a polyvinylidene difluoride membrane (Bio‐Rad Laboratories) and then incubated with anti‐STAT3 antibody (1:1,000; CST, Boston, MA), anti‐phospho‐STAT3 antibody (1:1,000; CST), and anti‐phosphatase and tensin homolog (PTEN) antibody (1:1,000; CST) at 4°C overnight. After incubation with the Horseradish peroxidase (HRP)‐conjugated antibody (CST), the membranes were visualized using an ECL Western Blotting Kit (Millipore).

### 2.8 Bioinformatic analysis of the miR‐21 promoter

Upstream regions were taken as 5,000 bases upstream and 100 bases downstream of the transcription start site (TSS). The identification of evolutionarily conserved transcription factor binding sites was performed using FIMO, a software tool for scanning DNA or protein sequences with motifs described as position‐specific scoring matrices. Secondary identification of transcription fact binding sites was performed using JASPAR and HOCOMOCO.

### 2.9 ChIP assay

HMECs were crosslinked with 1% formaldehyde, lysed, sonicated into small sequences, and then immunoprecipitated overnight with anti‐phospho‐STAT3 or anti‐rabbit immunoglobulin G (a negative control). The miR‐21 flanking genomic region containing STAT3‐binding sites was amplified by PCR using three primers: primer 1 sense, 5′‐TGCCTCCCAAGTTTGCTAATGC‐3′ and antisense, 3′‐ACAATCTGTGCGTCATCCTTATCC‐5′; primer 2 sense, 5′‐GCTTCCTGGGCTCTCACTGTAG‐3′ and antisense, 3′‐ GGTGGCTCACGCTTGTAATCC‐5′; and primer 3 sense, 5′‐AGTTCCTTGTGGGCAGTTTGG‐3′ and antisense, 3′‐ACATATCCCTAACTTCTGGCTGATTC‐5′.

### 2.10 Reporter vector construction and luciferase assays

For cloning of the human miR‐21 promoter reporter construct, the 1760‐bp fragment of the human miR‐21 promoter (from −3240 to −4999 relative to the translation start site) containing wild‐type or mutated STAT3‐binding sites was amplified and then inserted into the pGL3 basic vector. The STAT3 was constructed into the pCDNA3.1 eukaryotic expression vector. The 3′‐UTR sequences of PTEN messenger RNA (mRNA; PTEN‐3′‐UTR‐Wt or PTEN‐3′‐UTR‐Mut) were amplified by PCR and cloned into the pGL3 vector (Promega, Madison, WI). Cells (HEK293T) were seeded in 24‐well plates and allowed to attach overnight. The next day, the cells were cotransfected with pGL3‐miR‐21‐promoter (or pGL3‐basic) and pcDNA3.1‐STAT3 (or pcDNA3.1) when they reached 70–80% confluence. After 48 hr, luciferase activity was measured using the Dual‐Luciferase Reporter Assay system (Promega) according to the manufacturer's instructions. *Renilla* luciferase activity was normalized to firefly luciferase activity.

### 2.11 In vivo Matrigel plug assay

All animal experimental procedures were approved by the Animal Ethics Committee of Third Military Medical University. The Matrigel plug assay was performed as previously described. Briefly, growth factor‐reduced Matrigel (500 μl; BD Biocoat, Corning, NY) containing PBS, AngII (100 nM), AngII (100 nM)+Stattic (50 µM), AngII (100 nM)+miR‐21 antagomir (10 nM/mice), miR‐21 antagomir NC (10 nM/mice), or VEGF (50 ng/ml) was subcutaneously implanted into the right flanks of the mice (C57BL/6, male, 6–8 weeks old). After 14 days, the animals were killed, and the Matrigel plugs were removed and imaged (Leica MZ10 F, Wetzlar, Germany), embedded in paraffin, and sectioned for hematoxylin and eosin (H&E) staining and CD31 staining.

### 2.12 Statistical analysis

The data are presented as the mean ± standard deviation. Statistical analyses were performed by unpaired Student's *t* test or one‐way analysis of variance followed by Bonferroni's post hoc test using GraphPad Prism 6.0 (GraphPad Software, San Diego, CA). *p* < 0.05 indicates a significant difference.

## 3 RESULTS

### 3.1 AngII contributes to HMEC angiogenesis

**Figure 1:**
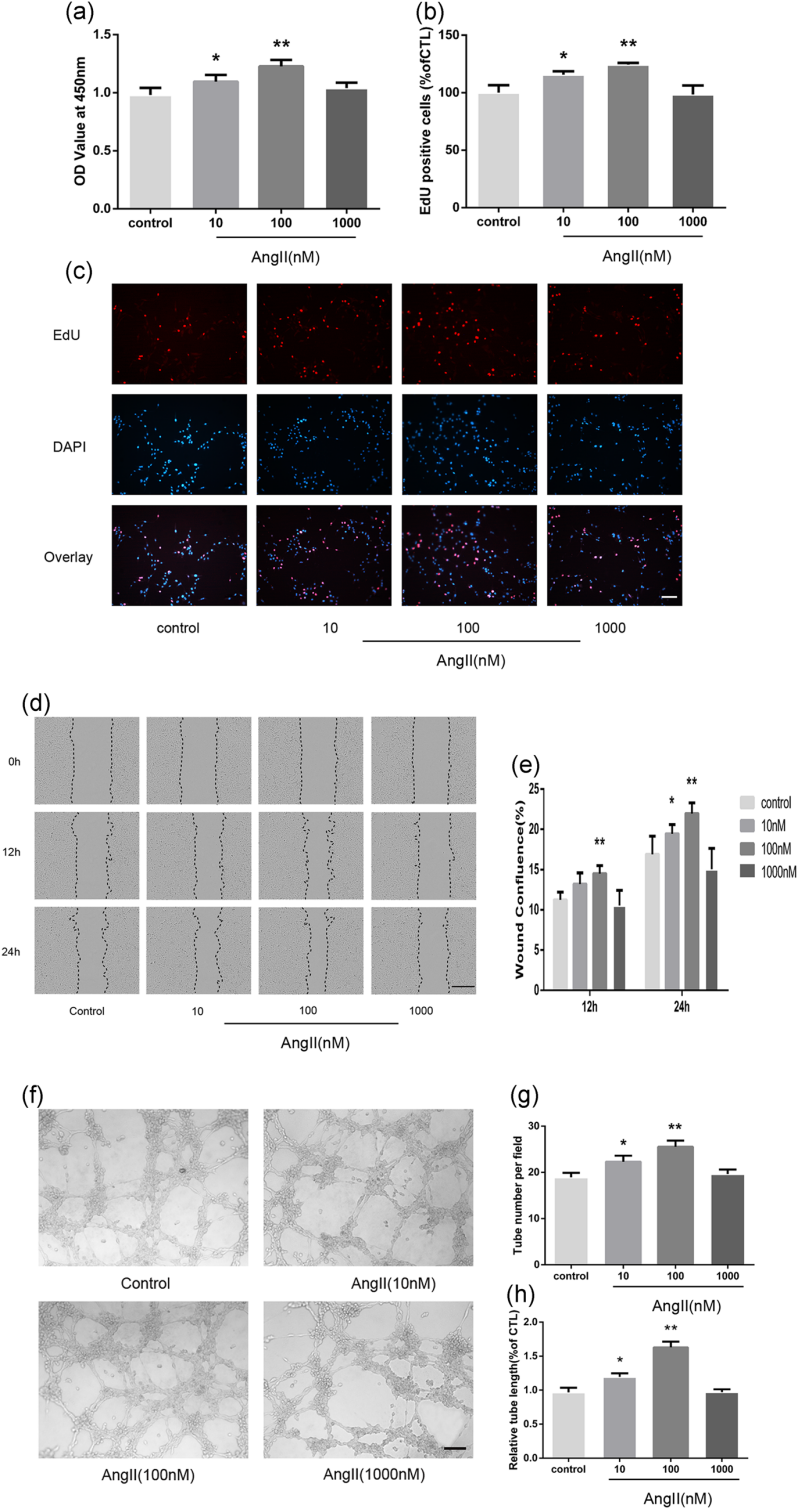
AngII induces human microvascular endothelial cell (HMEC) proliferation, migration, and capillary tube formation. (a) A Cell Counting Kit‐8 assay was performed to evaluate cell proliferation after 24 hr in corresponding serum‐free medium (*n* = 6). (b, c) EdU‐labeling analysis showed the fluorescence images of HMECs stimulated with 0, 10, 100, or 1,000 nM AngII for 24 hr (EdU, red fluorescent signals; DAPI, blue signals; ×100). (c) The percentage of DAPI‐positive cells indicated the quantification of EdU‐positive HMECs (*n* = 3; b). (d) HMECs monolayers were scratched and incubated with 0, 10, 100, or 1,000 nM AngII. Microscopic images were taken at 0, 12, and 24 hr using the IncuCyte ZOOM Live‐Cell Analysis System (Essen BioScience; ×40). (e) The relative wound confluence at 12 and 24 hr after wounding is shown (*n* = 3). (f) Representative micrographs of capillary tube formation by HMECs (1 × 10^4^/well) treated with AngII (0, 10, 100, 1,000 nM). (g, h) Mean numbers of capillary‐like tubes (g) and cumulative tube lengths (h) were quantified by the mean of the counts from five random fields (×100; *n* = 3). The data are presented as the mean ± standard deviation. *p < 0.05 vs. con. **p < 0.01 vs. con. Scale bar in Figure 1c,f represents 80 μm and in Figure 1d represents 400 μm

New blood vessel formation necessitates EC activation, proliferation, and migration to extend the pre‐existing vessels (D'Amore & Thompson, 1987). Thus, we first examined the physiological responses of HMECs to various concentrations of AngII. The proliferative effect of AngII was confirmed using CCK‐8 and EdU staining assays. As shown in Figure 1a–c, AngII promoted HMEC proliferation at concentrations of 10 and 100 nM; however, the induction was almost lost at a concentration of 1,000 nM. The scratch wound‐healing assay also showed that AngII enhanced endothelial cell migration at concentrations of 10 and 100 nM, with a maximum acceleration in the 100 nM group when determined at 24 hr (Figure 1d,e). However, no significant differences in HMEC mobility were observed between the control and 1,000 nM groups. Consistent with these results, HMECs treated with AngII at 10 and 100 nM showed a significant increase in tube formation, as demonstrated by the increased number of closed capillary tubes as well as their length (Figure 1f,g).

### 3.2 miR‐21 mediates AngII‐induced angiogenesis in HMECs

**Figure 2:**
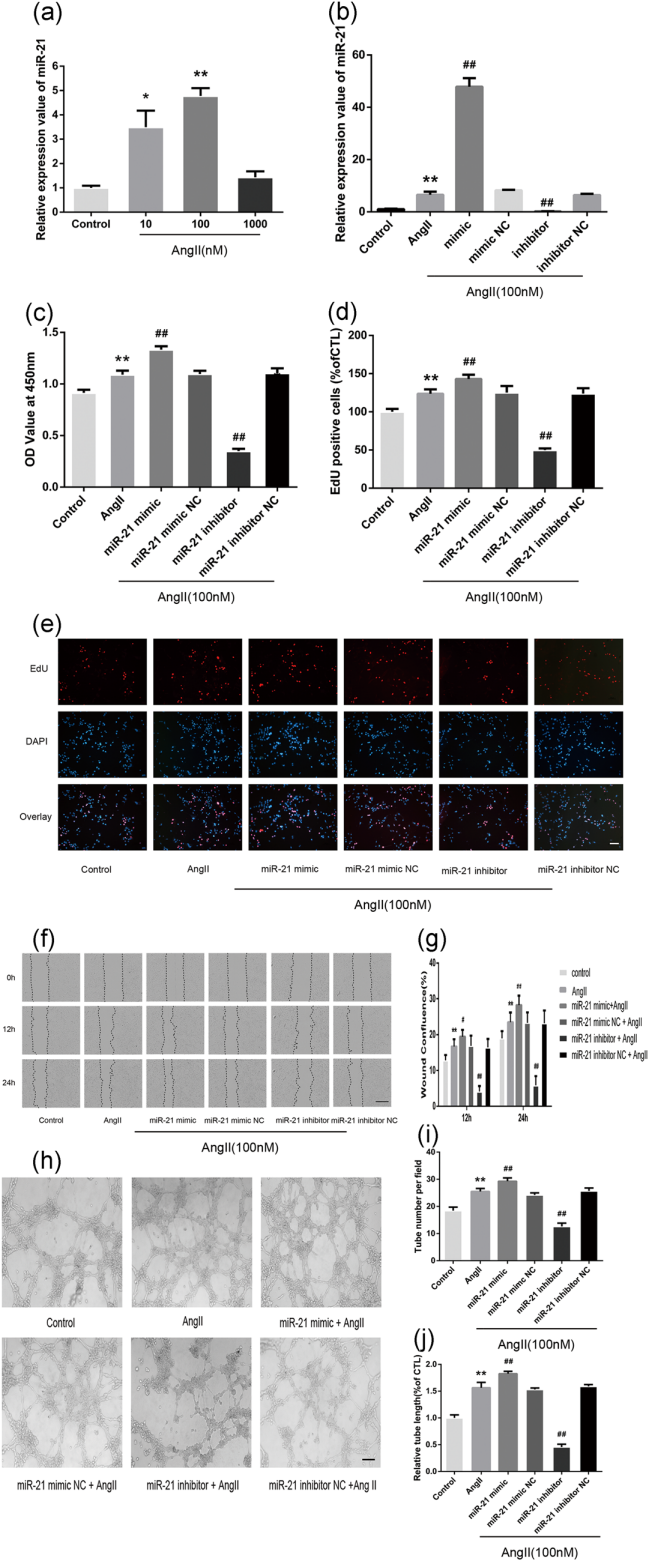
miR‐21 upregulation is associated with AngII‐induced angiogenesis in HMECs. (a) qPCR analysis of the relative expression value of miR‐21 in HMECs after incubation with different concentrations (0, 10, 100, and 1,000 nM) of AngII for 24 hr (*n* = 3). (b) miR‐21 expression in HMECs was detected by real‐time PCR after transfection (*n* = 3). (c) CCK‐8 assays were used to detect cell proliferation after transfection (*n* = 4). (d,e) Representative images of the EdU assay (e) and the quantified number of EdU‐positive cells. The miR‐21 inhibitor offset the AngII‐induced increase in HMEC proliferation, while the miR‐21 mimic exerted the opposite effect (d; *n* = 3). (f) Representative images of HMECs after wounding from the indicated experimental groups. (g) Relative wound confluence of HMECs 24 hr after the transfection of hsa‐miR‐21 mimic, hsa‐miR‐21 inhibitor, and their respective negative controls (*n* = 3). (h) Representative images portraying the formation of capillary‐like tubes in HMECs after the indicated transfections. (i,j) HMEC branch number and length. All data are presented as the mean ± standard deviation. *p < 0.05 vs. con. **p < 0.01 vs. con. ^#^ *p* < 0.05 vs. AngII. ^##^ *p* < 0.01 vs. AngII. The scale bars in Figure 2e,h represent 80 μm and in Figure 2f represent 400 μm. AngII: angiotensin II; CCK‐8: cell counting kit‐8; HMEC: human microvascular endothelial cell; miR‐21: miRNA‐21; qPCR: quantitative PCR

To determine whether miR‐21 plays a role in AngII‐induced angiogenesis, we evaluated the miR‐21 expression in HMECs after AngII treatment. Real‐time PCR results revealed that AngII treatment increased miR‐21 expression in HMECs at concentrations of 10 and 100 nM, and the expression was higher at 100 nM than at 10 nM (Figure 2a). As shown in Figure 2b, transfection with the miR‐21 mimic significantly elevated miR‐21 expression in HMECs; in contrast, transfection with the miR‐21 inhibitor decreased miR‐21 levels. Then, the effects of miR‐21 on cell proliferation, migration, and tube formation were examined using the miR‐21 mimic and inhibitor. In both the CCK‐8 and EdU tests, the AngII‐induced proliferation in HMECs was enhanced by the miR‐21 mimic but abolished by the miR‐21 inhibitor (Figure 2c–e). Meanwhile, the miR‐21 mimic obviously strengthened the AngII‐induced cell migration of HMECs, which was abrogated by the miR‐21 inhibitor (Figure 2f,g). Furthermore, after AngII treatment, HMECs transfected with the miR‐21 mimic showed an increase in capillary‐like tube formation, whereas miR‐21 inhibition suppressed the tube formation induced by AngII (Figure 2h–j).

### 3.3 AngII promotes angiogenesis in HMECs via STAT3 phosphorylation

**Figure 3:**
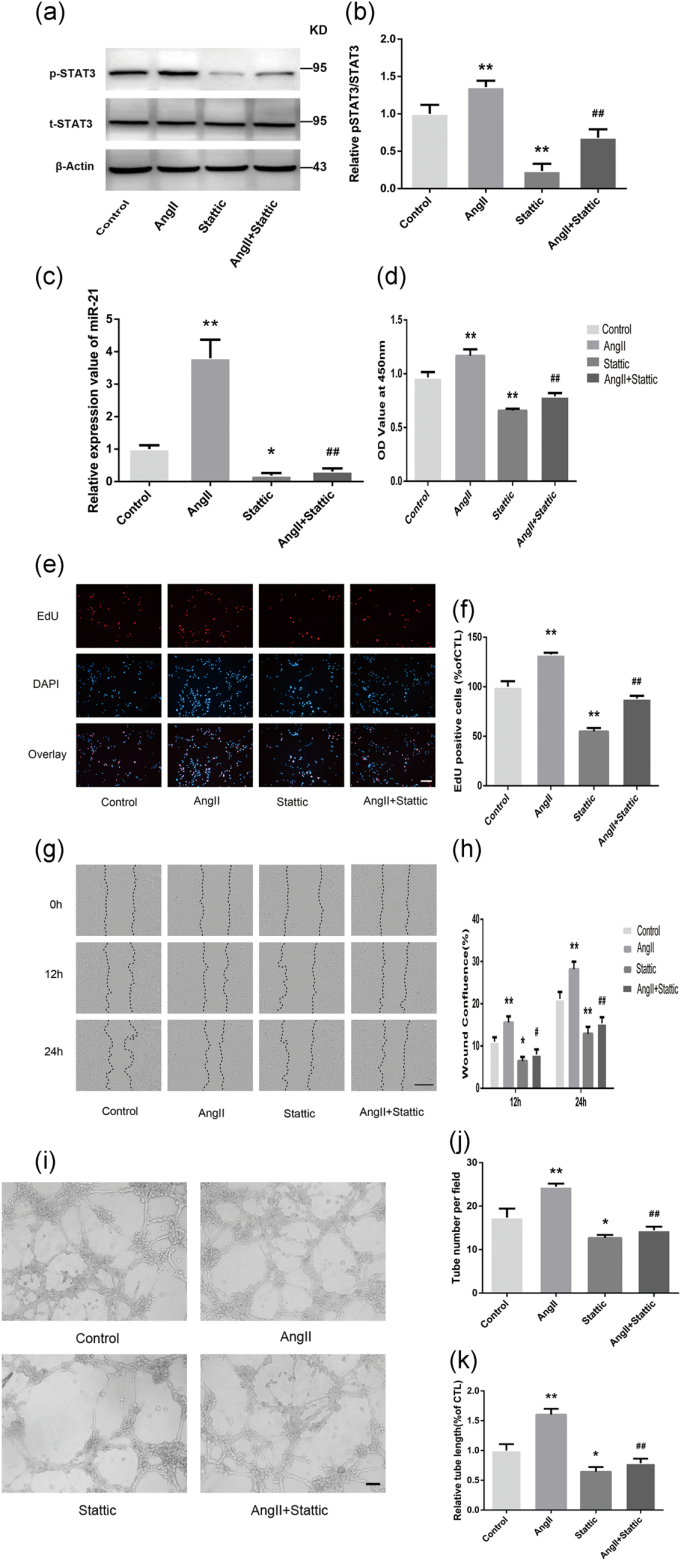
STAT3 phosphorylation is involved in AngII‐induced angiogenesis in HMECs. The HMECs were pretreated with Stattic (2 μM), a selective inhibitor, for 2 hr and then treated with AngII (100 nM) for 15 min. Western blot (a) and quantitative analyses (b) were performed to measure t‐STAT3 and p‐STAT3 (Tyr705) protein levels in HMECs (*n* = 3). (c) miR‐21 expression in HMECs was detected by real‐time PCR after AngII (24 hr) or Stattic treatment (2 hr; *n* = 4). (d) CCK‐8 assays were performed to detect HMEC proliferation (*n* = 4). Representative EdU images (e) and quantitative data (f) showing that HMEC cell proliferation was increased by AngII (100 nM), whereas these effects were reversed by coincubation with Stattic (2 µM; *n* = 3). (g,h) A scratch wound‐healing assay was conducted in HMECs with the indicated treatments. The migration distance was measured at 0, 12, and 24 hr after the cells had been scratched (*n* = 3). (i–k) HMECs were pretreated with Stattic (2 μM) for 2 hr and cocultured with or without AngII (100 nM). Representative images of in vitro angiogenesis assays (i). The tube number (j) and length (k) of each treatment are shown (*n* = 3). All data are presented as the mean ± standard deviation. *p < 0.05 vs. con. **p < 0.01 vs. con. ^#^ *p* < 0.05 vs. AngII. ^##^ *p* < 0.01 vs. AngII. The scale bars in Figure 3e,i represent 80 μm. In Figure 3g, the bar represents 400 μm. AngII: angiotensin II; CCK‐8: cell counting kit‐8; HMEC: human microvascular endothelial cell; miR‐21: miRNA‐21; STAT3: signal transducer and activator of transcription 3

Previous studies have demonstrated that the phosphorylation of STAT3 on Tyr705 is associated with the proangiogenic activity of STAT3 (Xu et al., 2016), which could be activated by AngII. However, whether AngII promotes angiogenesis via STAT3 has not been fully elucidated. Moreover, given that STAT3 is an important nuclear transcriptional factor, whether STAT3 mediated AngII‐induced miR‐21 expression was investigated. In HMECs, although total STAT3 protein levels remained unchanged, STAT3 phosphorylation was elevated almost immediately after AngII treatment (15 min; Figure 3a,b), consistent with previous reports of STAT3 activation in minutes to hours after the initial activation of AT1R (Marrero et al., 2004; Schmitz et al., 1998). This phosphorylation was effectively blocked by Stattic, a specific inhibitor of phosphorylated STAT3. As expected, the inhibition of STAT3 phosphorylation by Stattic resulted in a drastic reduction in miR‐21 expression induced by AngII exposure (Figure 3c). Given that previous results identified miR‐21 as the mediator for the proangiogenic role of AngII, it is reasonable that Stattic coadministration subsequently decreased AngII‐induced proliferation (Figure 3d–f), migration (Figure 3g,h), and tube formation (Figure 3i–k) in HMECs.

### 3.4 Identification of highly conserved STAT3‐binding sites in the proximal promoter region of miR‐21

**Figure 4:**
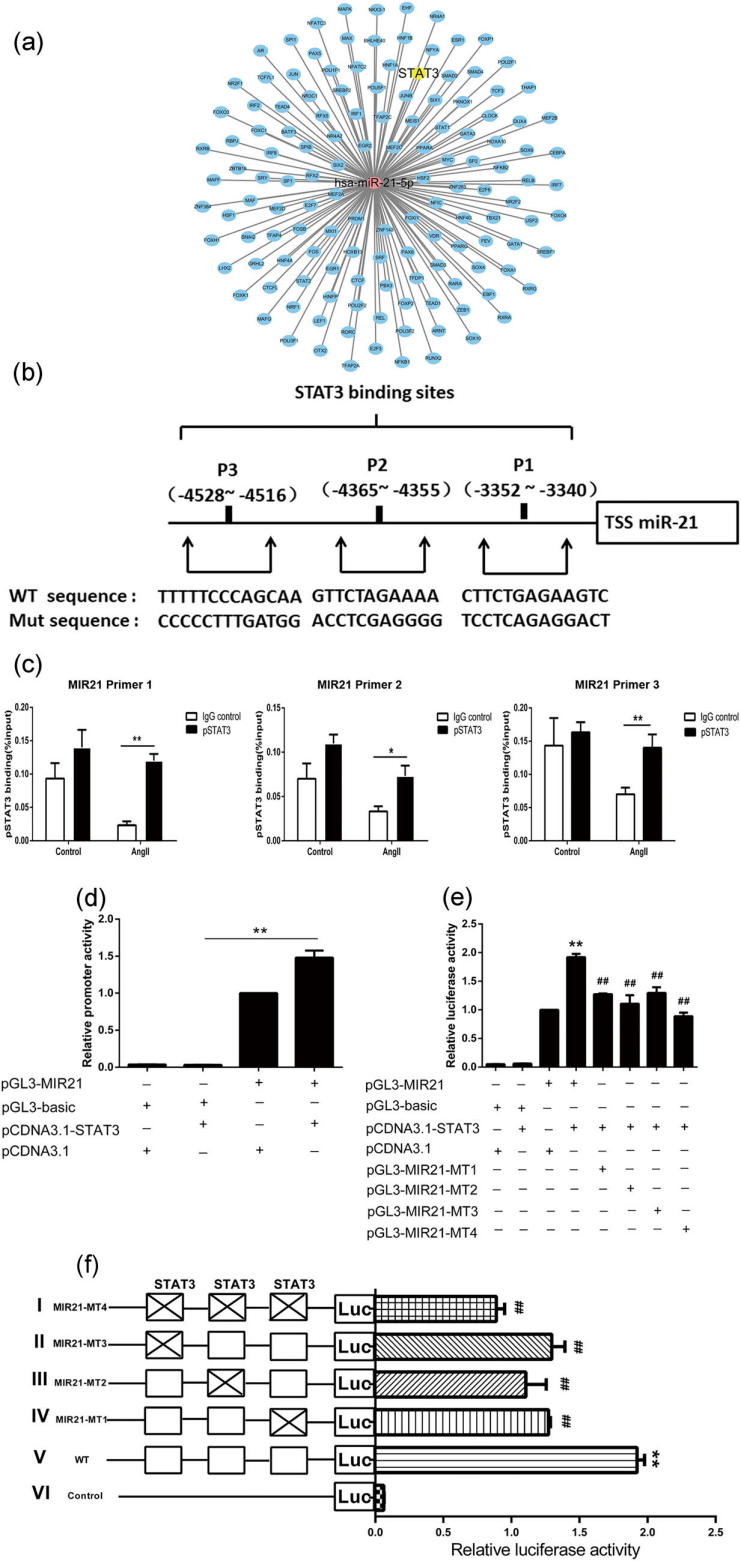
Three functional STAT3‐binding sites are identified in the miR‐21 proximal promoter region. (a) Bioinformatic analysis network of the transcription factors interacting with the promoter of miR‐21. The red circle represents hsa‐miR‐21‐5p, and the blue circles represent transcription factors related to essential biological processes. (b) Putative STAT3‐binding sites from the −3352 to −3340 positions, −4365 to −4355 positions, and −4528 to −4516 positions upstream of the miR‐21 transcription start site (+1) on miR‐21. (c) ChIP assays were performed to detect the binding of the p‐STAT3 protein to the miR‐21 promoter. Rabbit anti‐p‐STAT3 antibody or control rabbit IgG was used for immunoprecipitation with DNA isolated from HMECs. The immunoprecipitate was amplified by qPCR using primers targeting miR‐21. The results were normalized to the negative control IgG (*n* = 3). (d) Reporter gene assay using the miR‐21 promoter (*n* = 5). HEK293T cells were transfected with the indicated vectors for 48 hr; luciferase activities were measured with the Dual‐Luciferase Reporter System. (e) Promoter reporter assay of a firefly luciferase vector driven by miR‐21 (pGL3‐miR‐21‐Luc) containing the three STAT3‐binding sites shown in (b; *n* = 5). (f) Constructs containing mutations in all of the three binding sites (I), a single binding site (II, III, IV), or wild‐type (V) were cotransfected into HEK293T cells with a *Renilla* luciferase construct for normalization. The pGL3‐basic construct containing no promoter element (VI) was also transfected as a control. *p < 0.05 vs. IgG con, **p < 0.01 vs. IgG con in (c). **p < 0.01 vs. pGL3‐basic and pCDNA3.1‐STAT3 cotransfected group in (d). **p < 0.01 vs. pGL3‐miR‐21 and pCDNA3.1 cotransfected group. ^##^ *p* < 0.01 vs. pGL3‐miR‐21 and pCDNA3.1‐STAT3 cotransfected group in (e). **p < 0.01 vs. pGL3‐basic and pCDNA3.1‐STAT3 cotransfected group. ^##^ *p* < 0.01 vs. pGL3‐miR‐21 and pCDNA3.1‐STAT3 cotransfected group in (f). HMEC: human microvascular endothelial cell; IgG: immunoglobulin G; miR‐21: miRNA‐21; qPCR: quantitative PCR; STAT3: signal transducer and activator of transcription 3 [Color figure can be viewed at wileyonlinelibrary.com]

Despite the regulatory effect of STAT3 on miR‐21, whether STAT3 controls miR‐21 expression indirectly via other molecular signals or directly by binding to the miR‐21 promoter is unknown. Bioinformatic analysis identified dozens of potential regulatory transcription factors of miR‐21 based on its promoter sequence (Figure 4a). STAT3 was shown to be a potential transcription factor that might regulate miR‐21 expression. In silico analysis further revealed three putative evolutionarily conserved STAT3‐binding sites approximately 5 kb upstream of the TSS of the miR‐21 gene promoter region, referred to as P1 (−3352, −3340), P2 (−4365, −4355), and P3 (−4528, −4516; Figure 4b). The direct binding of p‐STAT3 to the miR‐21 gene promoter was further confirmed utilizing both ChIP‐qPCR assays and a dual‐luciferase reporter system (Figure 4c,d). Then, the specific binding site of STAT3 in the miR‐21 promoter was investigated by mutating the P1, P2, and P3 sites (Figure 4e,f). The results showed that the mutations in P1, P2, and P3 decreased the fluorescence intensity produced by STAT3 binding to regulatory elements of the miR‐21 promoter. Meanwhile, the luciferase activity of the miR‐21‐promoter‐driven luciferase reporter was substantially decreased when the three sites were mutated simultaneously in the presence of STAT3 (Figure 4e,f). These results thus indicated that STAT3 enhanced the transcriptional level of miR‐21 by directly binding to the promoter region on a sequence between −4528 and −3340 upstream of the TSS.

### 3.5 miR‐21 downregulates PTEN expression in HMECs

**Figure 5:**
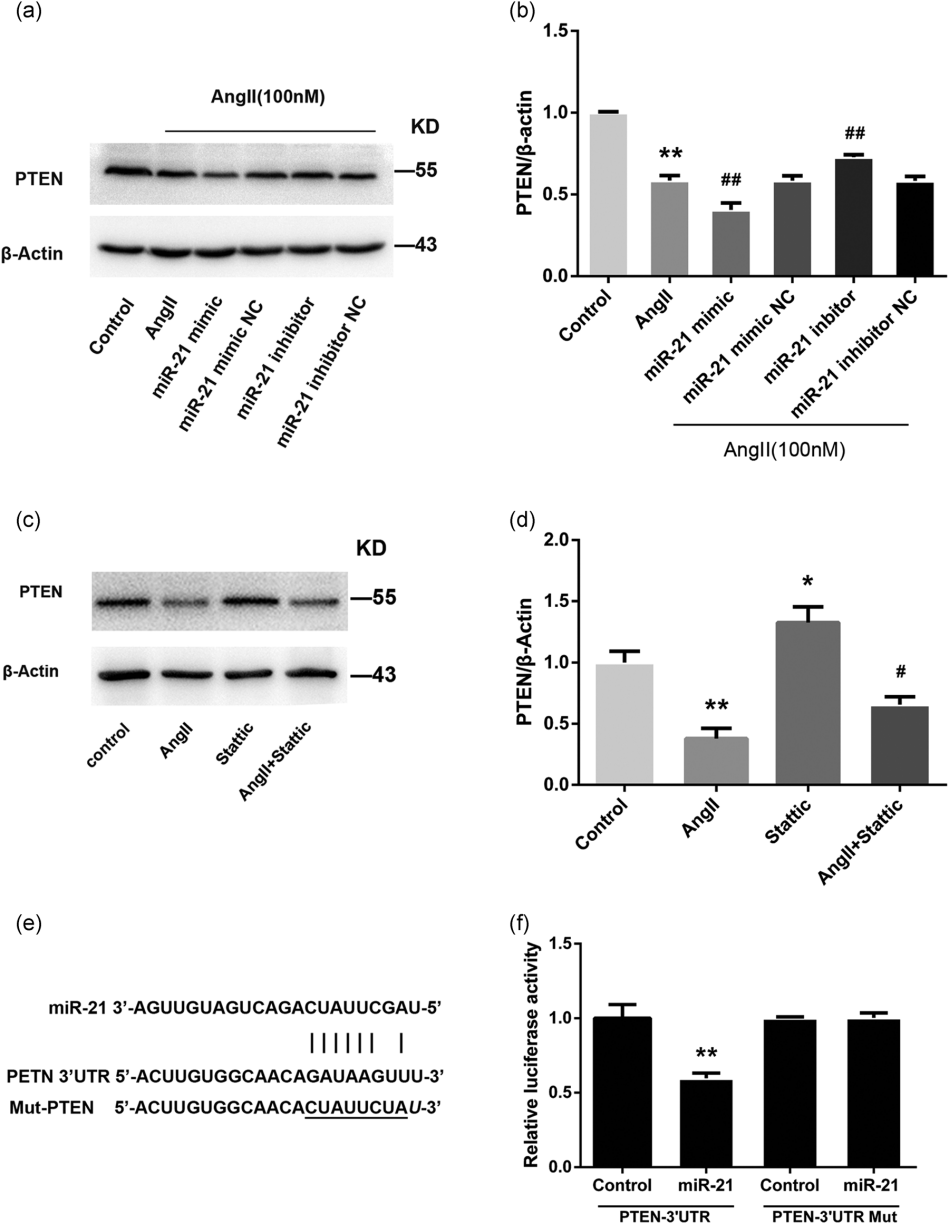
miR‐21 reduces PTEN expression by directly targeting its 3′‐UTR. (a,b) Representative western blots and quantitative analysis showing PTEN protein levels after transfection with miR‐21 mimic (30 nM), miR‐21 inhibitor (100 nM), or negative control (*n* = 3). (c,d) The effect of the STAT3‐specific inhibitor (Stattic) on the protein expression level of PTEN was determined by western blotting (c), and band intensities were quantified by optical density scanning (*n* = 3) (d). (e) Schematic illustration of reporter plasmid construction containing full‐length 3′‐UTR of PTEN. (f) An miR‐21 mimic (30 nM) or negative control was cotransfected with the luciferase reporter vector into HEK293 cells. The relative luciferase activity is shown (*n* = 3). All data are presented as the mean ± standard deviation. *p < 0.05 vs. con. **p < 0.01 vs. con. ^#^ *p* < 0.05 vs. AngII. ^##^ *p* < 0.01 vs. AngII. AngII: angiotensin II; miR‐21: miRNA‐21; PTEN: phosphatase and tensin homolog

Several studies have identified PTEN as a target of miR‐21 that mediates various pathophysiological processes, including angiogenesis (Luo et al., 2017; J. L. Mehta et al., 2015). However, whether AngII‐induced miR‐21 targets PTEN requires further verification. As shown in the immunoblotting results, AngII led to a decrease in PTEN protein, which was enhanced by the miR‐21 mimic and attenuated by the miR‐21 inhibitor (Figure 5a,b). Moreover, the AngII‐mediated reduction in PTEN was reversed by the STAT3 inhibitor Stattic (Figure 5c,d), further clarifying the role of PTEN as a mediator of the AngII/STAT3/miR‐21 signaling pathway. We then investigated whether miR‐21 directly recognizes the 3′‐UTR of PTEN mRNA using the dual‐luciferase reporter assay. As shown in Figure 5e,f, the reporter activity for wild‐type PTEN‐3′‐UTR was significantly reduced by miR‐21 transfection, whereas the reporter activity for the mutated PTEN‐3′‐UTR was unaffected.

### 3.6 Blockade of the STAT3/miR‐21 pathway attenuates AngII‐induced angiogenesis in vivo

**Figure 6:**
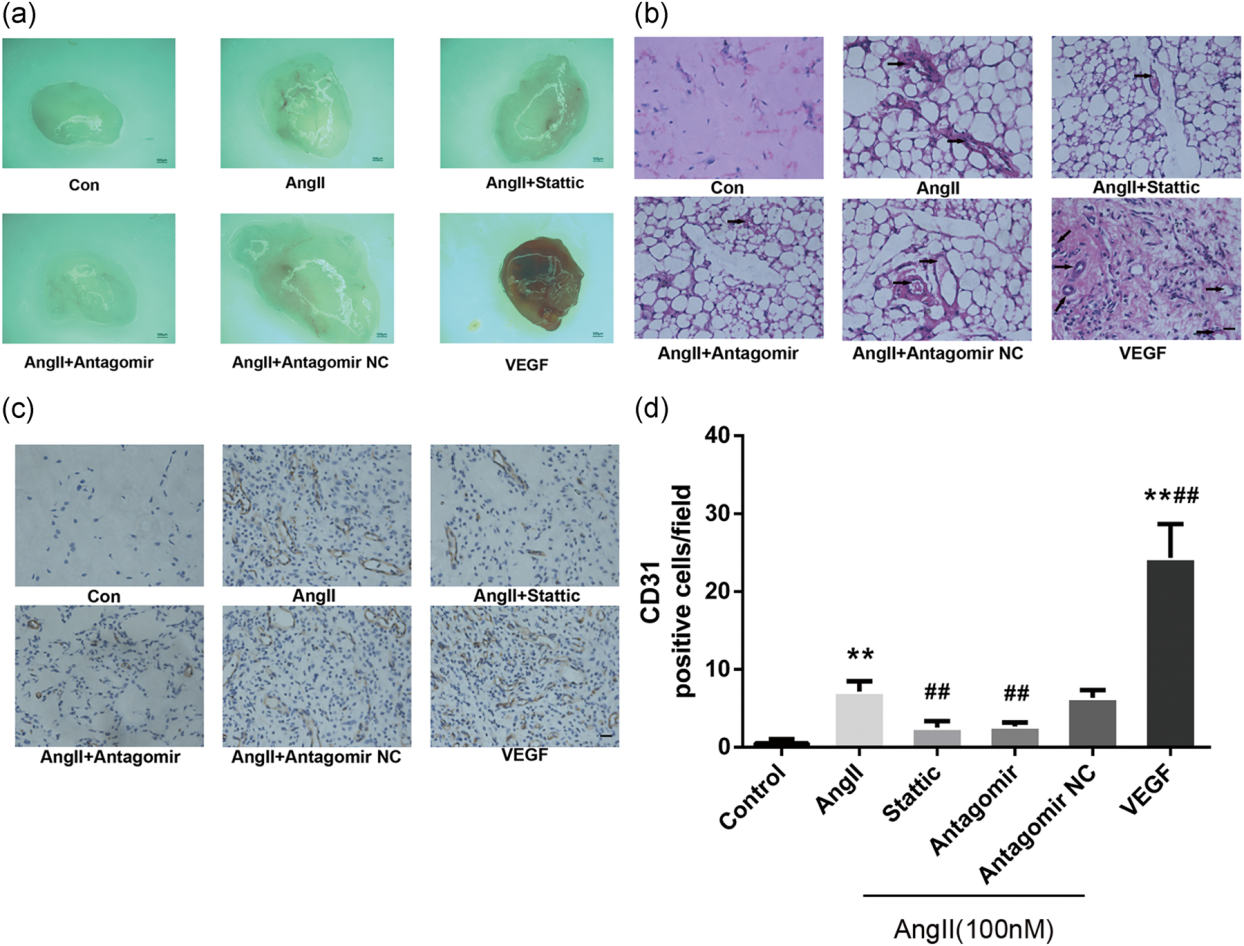
The inhibition of STAT3/miR‐21 pathway abrogated AngII‐induced angiogenesis in vivo. A Matrigel mixture containing VEGF (50 ng/ml) and AngII (100 nM) alone or in combination with either Stattic (50 µM) or miR‐21 antagomir (10 nM) was injected subcutaneously into the ventral side of the mice. After 14 days, the Matrigel plugs were recovered. Each group consisted of six mice. (a) Representative images of macroscopic visualization of Matrigel plugs at 14 days. (Leica MZ10 F; Leica, Wetzlar, Germany), ×80. (b, d) Plugs from implanted mice were subjected to H&E staining (b) and immunohistochemical staining for CD31 (c, d); representative images (c) for quantitative analysis (d; *n* = 6). Scale bars: 500 μm in (a), 19.6 μm in (b) and (c). New vessel formation (black arrowheads) was observed at ×400. *p < 0.05 vs. con. **p < 0.01 vs. con. ^#^ *p* < 0.05 vs. AngII. ^##^ *p* < 0.01 vs. AngII. AngII: angiotensin II; H&E: hematoxylin and eosin; STAT3: signal transducer and activator of transcription 3 [Color figure can be viewed at wileyonlinelibrary.com]

To further evaluate the proangiogenic effect of AngII and the roles of STAT3 and miR‐21 in vivo, we used a well‐established murine model for Matrigel plug neovascularization assays. After 14‐day subcutaneous implantation of a Matrigel mixture, the Matrigel plug containing AngII alone presented more extensively distributed neovascularization compared with that of the control group (PBS; Figure 6a). In contrast, following the addition of Stattic, the proangiogenic response to AngII was reversed (Figure 6a). Meanwhile, in the group treated with the combination of AngII and antagomir of miR‐21, there was a small number of new blood vessels (Figure 6a). Using H&E staining and immunohistochemical staining with CD31, we determined the regional microvascular density of the Matrigel mixture (Figure 6b–d). Similar results were obtained with a specific inhibitor of STAT3, which suppressed AngII‐induced angiogenesis. In addition, the enhanced angiogenesis by AngII was diminished by the combination with the miR‐21 antagomir.

## 4 DISCUSSION

AngII has been reported to affect multiple pathophysiologic processes, which might be closely related to cardiovascular diseases and cancer (Penafuerte et al., 2016; Phie et al., 2018; Ranjbar et al., 2018). AngII can promote angiogenesis in vivo and in vitro via the activation of the AT1R, which might be associated with the progression and instability of atherosclerotic plaques (Daugherty, Manning, & Cassis, 2000; Le Noble et al., 1993; Munzenmaier & Greene, 1996; Tamarat, Silvestre, Durie, & Levy, 2002). Several studies have reported that AngII may exert potent proangiogenic activity by upregulating other angiogenic factors, including VEGF‐A, basic fibroblast growth factor, platelet‐derived growth factor, insulin‐like growth factor 1, epidermal growth factor, and transforming growth factor β (Si et al., 2018). However, it is largely unknown whether miRNAs and their transcription factors play crucial roles in the angiogenic process induced by AngII. In this study, we showed that STAT3 exhibited proangiogenic properties by binding directly to the miR‐21 promoter or regulatory region and initiating the transcription of miR‐21. We further demonstrated that the enhanced proliferation, migration, and tube formation capacity induced by AngII at least partly resulted from the suppressed expression of PTEN protein through the activation of miR‐21 pathway via STAT3.

It is generally considered that alterations in the growth and apoptosis pathways mediated by AngII play a pivotal role in the onset and development of atherosclerosis. However, it appears difficult to define the contribution of specific pathways in the regulation of atherosclerosis. Accordingly, additional information is required on the interplay between these pathways and the mediators of growth and apoptosis. We showed here that a moderate concentration of AngII has implicated the formation of new blood vessels in vivo and in vitro, which was consistent with the findings of previous studies (Hoffmann et al., 2017; Mai et al., 2014; Wu et al., 2014).

Recent studies have demonstrated that miR‐21, a well‐known miRNA widely studied in the pathogenesis of vascular inflammation and diseases, may be responsible for plaque progress by inducing the formation of new blood vessels in atherosclerotic lesions (Jansen et al., 2015; Urbich, Kuehbacher, & Dimmeler, 2008). Numerous studies have been conducted on the miR‐21‐regulated proliferation and migration of ECs; however, the results remain controversial. While Sabatel et al. (2011) reported that miR‐21 overexpression decreased the angiogenic activity of HUVECs, Guduric‐Fuchs et al. (2012) reported that the inhibition of miR‐21 disturbed the formation of vascular networks in retinal microvascular endothelial cells. These conflicting results were mainly due to cell type‐specific responses to various extracellular stimuli. Indeed, we revealed that the induction of miR‐21 expression by AngII was reversed by miR‐21 inhibitor coincubation, followed by a reduction in proliferation viability, mobility, and capillary tube‐like formation activity of HMECs.

Transcription factors regulate the expression of genes (including protein‐coding genes and miRNA precursors) by binding to a specific DNA sequence that may either positively or negatively regulate a variety of biological processes, such as cell division, cell growth, and cell death, in response to external stimuli. In the cardiovascular system, STAT3 is a key target for AngII signaling and is an extremely pivotal mediator of cardiovascular remodeling (Beak et al., 2019; Ye et al., 2017). Once activated by cytokines such as interleukin‐6 through phosphorylation at Tyr705 (pY705) or Ser727, phosphorylated STAT3 is upregulated (Murase & McKay, 2014). Moreover, the altered p‐STAT3 expression is associated with angiogenesis and has been considered a new therapeutic target to inhibit tumor growth and angiogenesis (Banerjee & Resat, 2016; Bhat et al., 2013). The AngII‐activated STAT3 signaling cascade in various cell types has been shown to induce intracellular signal transduction and regulates a number of cellular processes involved in cell proliferation, cell survival, cell adhesion, and inflammation (Brands et al., 2010; Yang et al., 2018). Recent studies have revealed that STAT3 could induce the upregulation of miR‐21 (Chen et al., 2018; Su et al., 2017), whereas Jiang et al reported that the expression of phosphorylated STAT3 was concomitantly decreased by a miR‐21 inhibitor, indicating that there may be a feedback loop among miR‐21 and p‐STAT3 (Jiang et al., 2015). Meanwhile, in our study, we determined the regulatory effect of STAT3 on miR‐21 expression after AngII exposure in HMECs. Consistent with the findings of a previous study, we observed that pretreatment with AngII (100 nM) for 15 min resulted in a significant increase in the Tyr705 phosphorylation of STAT3 (Kandalam & Clark, 2010). At the same time, we found that the proangiogenic activity of AngII was related to the phosphorylation level of STAT3 because the addition of a specific inhibitor of STAT3 phosphorylation (Stattic) diminished AngII‐induced proliferation, migration, and tubular network formation on Matrigel. We also identified novel STAT3‐binding sites (P1 −3352, −3340, P2 −4365, −4355, and P3 −4528, −4516) in an evolutionarily conserved region upstream of miR‐21. Our ChIP and reporter gene assay results further indicated that STAT3 could directly induce the transcriptional activity of miR‐21, which showed that STAT3/miR‐21 could cooperatively promote angiogenesis development. Thus, we proposed a role of STAT/miR‐21 axis in EC angiogenesis and atherogenesis in response to an unfavorable stimulus such as AngII exposure.

Numerous studies have shown that AngII‐mediated promotion of proliferation of other cell types is regulated by induction of miR‐21, which negatively modulates the expression of PTEN, Smad7, and Sprouty1 (Ning et al., 2017; Schmitz et al., 1998). The PTEN gene was inactivated by several mechanisms, such as genetic mutation, promoter methylation, and posttranscriptional modification, which might contribute to tumor cell proliferation, migration, and increased numbers of microvessels in tumors and eventually lead to the malignant progression of the tumor (Serra et al., 2015). In this study, we observed a significant negative correlation between miR‐21 and PTEN protein in HMECs. Our results are further supported by previous studies, which have revealed that the 3′‐UTR of PTEN is directly targeted by miR‐21 in different cell types. Thus, we suggest that PTEN is a critical mediator in the development of atherosclerosis.

AngII is reported to exert dual biologic actions, which might largely depend on receptor/ligand affinity, alteration in trafficking patterns, AT1R structural modifications, and the local tissue environment. This molecule functions in the endocrine system, but it also serves local paracrine and autocrine functions in tissues and organs (P. K. Mehta & Griendling, 2007). Moderate concentrations of AngII provoke cell proliferation and migration, whereas high concentrations induce apoptosis and growth arrest. Consequently, the local cellular responses to dynamic AngII concentrations in atherosclerotic plaques may stimulate inflammatory or anti‐inflammatory signals or proangiogenic or proapoptotic responses, thereby contributing to plaque growth, instability, and rupture. These dual properties of AngII are involved in every step of atherosclerosis pathogenesis, from early fatty streak formation to late advanced atherosclerotic complex lesions. Once exposed to moderate concentrations of AngII, ECs grew from the existing adventitial vasa vasorum to form immature, irregular, and fragile neovessels in vulnerable plaques, subsequently contributing to IPA, IPH, and the progression of advanced atherosclerotic plaques. Therefore, we established a model here for the role of activation of the STAT3/miR‐21 axis, which, in turn, suppressed PTEN protein expression and, thus, finally promoted EC angiogenesis. Thus, pharmacological inhibition of AngII appears to prevent the occurrence and progression of atherosclerosis. Our study provides novel insights into the angiogenic activity induced by AngII.

We propose that studying active transcription factor‐miRNA transcriptional regulatory networks, such as STAT3‐miRNA networks, in specific vascular endothelial cell types can help further elucidate the regulation of miRNAs, the gene networks and the cellular pathways between angiogenesis and atherosclerosis and perhaps lead to the development of pharmacologically novel therapeutic approaches for improved antiangiogenic strategies to prevent the progression and instability of atherosclerotic plaques.

**Figure 7:**
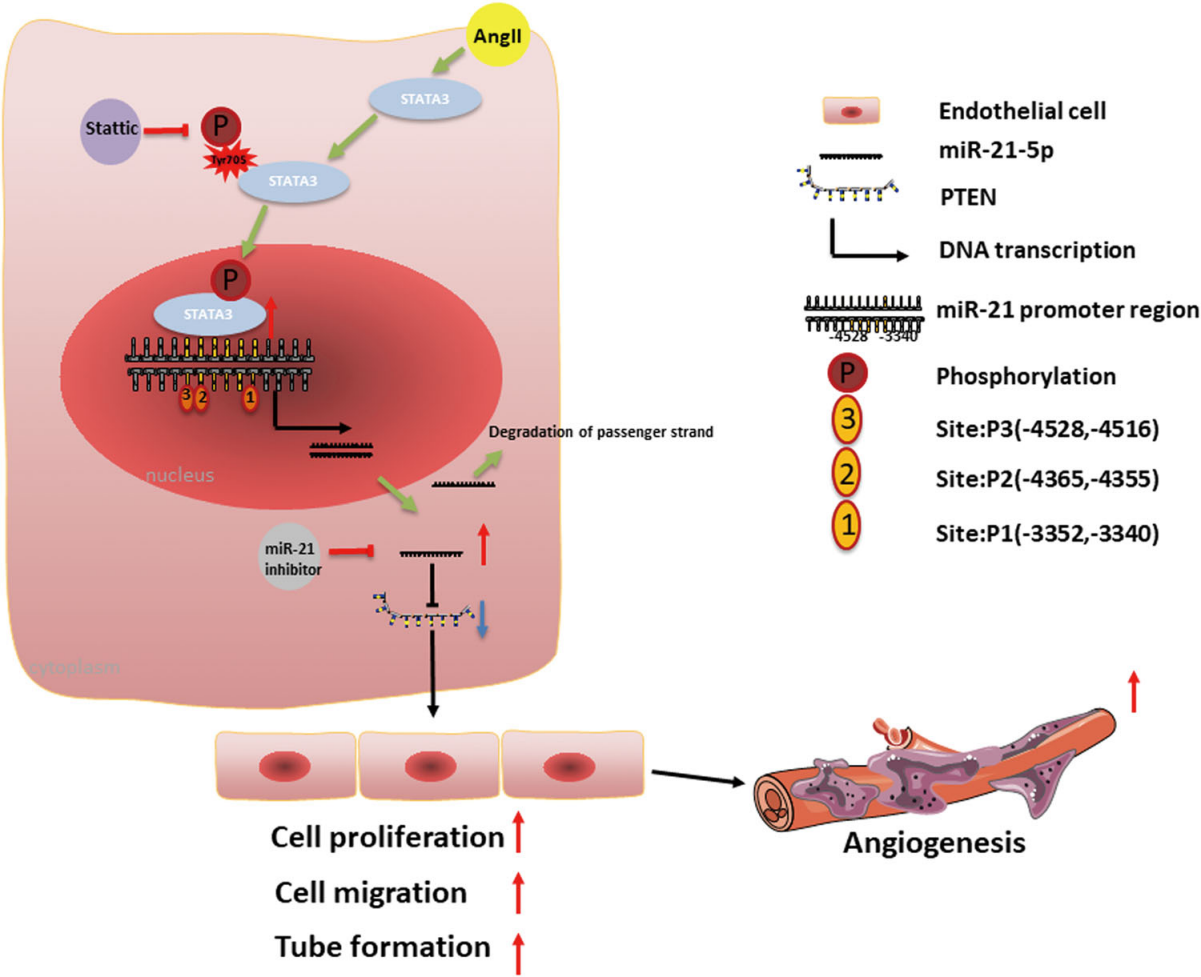
Schematic diagram of the proposed mechanism for AngII‐induced angiogenesis. AngII upregulates miR‐21 expression in ECs by activating STAT3. Upregulation of miR‐21 by AngII inhibits PTEN expression by directly targeting the 3′‐UTR, leading to increased proliferation, migration, and tube formation in ECs. Targeting the STAT3/miR‐21 axis decreases AngII‐induced angiogenesis in vivo and in vitro. AngII: angiotensin II; EC: endothelial cell; PTEN: phosphatase and tensin homolog; STAT3: signal transducer and activator of transcription 3 [Color figure can be viewed at wileyonlinelibrary.com]

In summary, as shown in the diagram in Figure 7, our study demonstrated that the STAT3/miR‐21 pathway was involved in AngII‐induced angiogenic sprouting in HMECs. We further revealed that STAT3‐mediated inhibition of PTEN via the induction of miR‐21 in HMECs exposed to AngII is a part of the epigenetic switch linking angiogenesis to atherosclerosis. Our results indicated that targeting the STAT3/miR‐21 axis in combination with existing conventional strategies may serve as effective therapies for atherosclerosis.

## CONFLICT OF INTERESTS

The authors declare that there is no conflict of interests.

## AUTHOR CONTRIBUTIONS

H. Y. H., Y. H. L., and L. Y. C. conceived and designed research; L. Y. C., X. W., X. L. Q., L. N. P., and Z. Y. W. performed research; L. Y. C., X. W., and X. L. Q. analyzed data; L. Y. C. drafted the manuscript; H. Y. H. and Y. H. L. reviewed the manuscript.
